# Host Immunogenetic Profile Modulates Susceptibility to Apical Periodontitis in a Colombian Population

**DOI:** 10.3390/cimb48010107

**Published:** 2026-01-20

**Authors:** Ingrid Giraldo-Quiceno, Natalia Andrea Torres-Calvo, Andrés Felipe Ayala-Jaramillo, Christina Garcés, Sandra Catalina Garzón-Castaño, Beatriz Giraldo-Ospina, Nora Elena Valencia-Marroquín, Carlos Manuel Beltrán-Díaz, Iván Alberto Lopera-Castrillón, Carlos Andrés Naranjo-Galvis

**Affiliations:** 1 Facultad de Salud, Universidad Autónoma de Manizales, Antigua Estación del Ferrocarril, Manizales 170004, Colombia; 2 Grupo de Investigación Biomedicina, Facultad de Medicina, Institución Universitaria Visión de las Américas, Pereira 660004, Colombia; 3 Grupo de Investigación GIUR, Vicerrectoria de Investigación y Proyección Social, Unidad Central del Valle del Cauca, Tuluá 763022, Colombia; 4 Grupo de Investigación GIECSA, Facultad de Ciencias de la Salud, Unidad Central del Valle del Cauca, Tuluá 763022, Colombia; 5 Grupo de Investigación Salud, Cuidado y Sociedad, Facultad de Ciencias de la Salud, Unidad Central del Valle del Cauca, Tuluá 763022, Colombia

**Keywords:** apical periodontitis, genetic polymorphisms, *MMP1*, cytokines

## Abstract

Apical periodontitis (AP) is a chronic immunoinflammatory disease influenced by complex interactions between microbial factors and host immune response. Although genetic susceptibility has been implicated in AP, evidence is limited, particularly in admixed populations. This exploratory study aimed to assess whether functional polymorphisms in *MMP1* (rs1799750), *IL10* (rs1800872), and *IL17A* (rs7747909) are associated with susceptibility to radiographically defined AP in a Colombian population. A case–control design was employed, including individuals with radiographic evidence of AP and controls without periapical lesions. Genotyping was performed using TaqMan^®^ assay. The association between single-nucleotide polymorphisms and AP was evaluated using a dominant inheritance model. Effect sizes were estimated as odds ratios (ORs) with 95% confidence intervals (CIs), and *p*-values were adjusted using the Benjamini–Hochberg false discovery rate (FDR) procedure. The *MMP1* rs1799750 polymorphism was associated with increased susceptibility to AP (OR = 3.47, 95% CI = 1.40–8.58; FDR = 0.013). Similarly, the *IL10* rs1800872 variant was significantly associated with AP risk (OR = 3.00, 95% CI = 1.52–5.91; FDR = 0.007). The strongest association was observed for *IL17A* rs7747909 (OR = 8.95, 95% CI = 3.61–22.15; FDR < 0.001). This exploratory candidate-gene study provides preliminary evidence suggesting that genetic variations in *MMP1*, *IL10*, and *IL17A* may contribute to susceptibility to AP in the Colombian population. Given the exploratory design, modest sample size, and absence of ancestry adjustment or functional validation, these findings should be interpreted cautiously and confirmed in larger ancestry-informed cohorts integrating host genetic and microbial data.

## 1. Introduction

Apical periodontitis (AP) is a chronic immunoinflammatory disease of the periapical tissues that develops as a consequence of persistent microbial infection within the root canal system of teeth with necrotic pulp [1]. While bacterial biofilms constitute the primary etiological factor, increasing evidence indicates that the host immune response critically modulates disease susceptibility, lesion persistence, and tissue destruction [2,3]. Notably, individuals exposed to comparable microbial challenges may exhibit markedly different periapical outcomes, suggesting an important contribution of host-related factors beyond the microbial burden.

AP involves a dynamic interplay between innate and adaptive immune responses, including cytokine signaling, immune cell recruitment, and extracellular matrix remodeling [4]. These processes determine whether inflammation is effectively contained or progresses toward chronic bone resorption and lesion expansion. From this perspective, AP can be conceptualized as an immunoinflammatory condition influenced by interindividual variability in immune regulation and tissue remodeling capacity [5,6].

Recent advances in periodontal genomics have demonstrated that susceptibility to oral inflammatory diseases follows a polygenic architecture, with multiple genetic variants exerting modest effects that interact with environmental and behavioral factors [7,8,9]. Genome-wide association studies (GWAS) of periodontitis have consistently implicated loci related to cytokine signaling, innate immune recognition, and extracellular matrix degradation [10,11,12,13,14]. However, AP remains comparatively understudied at the genomic level, and the extent to which immune-regulatory genetic variants contribute to AP susceptibility has not been fully elucidated [6]. Most available studies rely on candidate-gene approaches, often with limited sample sizes and heterogeneous populations, underscoring the need for population-specific investigations [15].

Among the biologically plausible candidate genes, *MMP1*, *IL10*, and *IL17A* represent complementary components of inflammatory regulation and tissue remodeling pathways implicated in AP. *MMP1* encodes a collagen-degrading enzyme involved in extracellular matrix turnover and periapical bone remodeling, and its promoter polymorphism rs1799750 (−1607 1G/2G) has been associated with increased transcriptional activity [16,17]. In contrast, *IL17A* encodes a pro-inflammatory cytokine central to Th17-mediated immune responses, and regulatory variants such as rs7747909 have been linked to enhanced inflammatory signaling in oral and systemic inflammatory conditions [18,19,20]. *IL10* functions as a key anti-inflammatory mediator, and promoter variants including rs1800872 (−592 C>A) influence cytokine expression and the resolution of inflammation [15,21,22].

Importantly, genetic susceptibility to inflammatory diseases is strongly influenced by the ancestry of the population. Colombian populations exhibit a complex admixture of Native American, European, and African ancestries, resulting in allele frequency distributions and linkage patterns that differ from those observed in European and Asian populations [23,24]. This genetic heterogeneity highlights the importance of conducting population-specific studies to contextualize immunogenetic associations in AP.

Although direct genetic evidence linking specific polymorphisms to AP remains limited, systematic reviews and integrative analyses of periodontal and periapical diseases consistently highlight the central role of immune regulation and extracellular matrix remodeling in disease pathogenesis. In particular, dysregulation of matrix metalloproteinases, imbalance between pro- and anti-inflammatory cytokines, and activation of Th17-related pathways have been repeatedly implicated in periapical inflammation and bone destruction. These convergent lines of evidence provide a biologically grounded rationale for evaluating functional variants in *MMP1*, *IL10*, and *IL17A* as candidate susceptibility genes in AP, despite the current paucity of disease-specific genetic association studies.

Therefore, this study aimed to assess whether functional polymorphisms in *MMP1* (rs1799750), *IL10* (rs1800872), and *IL17A* (rs7747909) are associated with susceptibility to radiographically defined AP in a Colombian population. AP was conceptualized as a binary disease phenotype based on radiographic and clinical evidence of periapical pathology, irrespective of symptom presentation, as the study was not designed to stratify cases according to the symptomatic status.

Based on existing evidence implicating inflammatory regulation and extracellular matrix remodeling in periapical tissue pathology, we hypothesized that genetic variants associated with enhanced pro-inflammatory signaling (*IL17A*), reduced anti-inflammatory regulation (*IL10*), and increased extracellular matrix degradation (*MMP1*) may contribute to interindividual differences in susceptibility to apical periodontitis by reflecting an imbalance between inflammatory activation and resolution mechanisms. Given the candidate gene design, this hypothesis is biologically motivated but exploratory in nature and does not imply causality.

## 2. Materials and Methods

### 2.1. Study Design

This research employed a case–control study aimed to determine the association between selected genetic polymorphisms and susceptibility to AP. The scope of the study was correlational and explanatory, seeking to establish associations between independent variables (genetic variants in *MMP1*, *IL10*, and *IL17A*) and the dependent variable (presence or absence of AP), while controlling for demographic and clinical factors.

### 2.2. Ethical Considerations

This study was conducted in strict accordance with the ethical principles outlined in the Declaration of Helsinki and the national regulations for health research established by the Colombian Ministry of Health (Resolution No. 8430, 1993). The research protocol was reviewed and approved by the Institutional Ethics Committee of the institución de Educación Superior-Unidad Central del Valle del Cauca (UCEVA) (approval code: 10122021). Prior to enrollment, all participants (or their legal guardians, where applicable) received a comprehensive explanation of the study’s objectives, procedures, potential risks, and anticipated benefits and provided written informed consent. Participant privacy and data confidentiality were rigorously safeguarded through the use of anonymized identifiers and secure, password-protected databases accessible exclusively to authorized research team members.

### 2.3. Inclusion and Exclusion Criteria

Strict quality control procedures were implemented to ensure the methodological rigor and internal validity of this study. The clinical classification of the cases and controls was performed by calibrated examiners specializing in endodontic diagnosis to minimize inter-observer variability. Inter-examiner reliability was verified in a random subset of radiographic assessments, yielding a Cohen’s kappa coefficient of >0.85, which is indicative of excellent agreement. AP was diagnosed based on the presence of radiographically detectable periapical radiolucency and compatible clinical findings. For genetic association analysis, cases were classified as having AP regardless of symptom status (symptomatic or asymptomatic). Systematic stratification according to pain, swelling, or acute clinical presentation was not performed, and symptom-based subgroups were not analyzed separately.

The control group comprised individuals with vital teeth, no caries or periodontal disease, and no periapical pathology. Participants were excluded if they had systemic inflammatory or autoimmune conditions, metabolic disorders, recent (within the last three months) use of antibiotics or corticosteroids, pregnancy, or incomplete clinical or demographic data. These exclusion criteria were designed to reduce the confounding effects related to systemic inflammation and ensure homogeneity of the study population. Eligible participants were subsequently classified as cases or controls according to the diagnostic criteria described in Section 2.4.

### 2.4. Diagnostic Criteria and Case–Control Classification

Apical periodontitis (AP) was defined based on combined radiographic and clinical criteria. Radiographic evaluation was performed using periapical radiographs obtained as part of a routine dental assessment. Cases were defined as individuals presenting with at least one tooth with a periapical radiolucent lesion consistent with apical periodontitis, as determined by an experienced clinician. Radiolucent areas in the periapical region, indicative of the loss of normal periapical bone structure, were considered diagnostic of AP.

AP classification was based on radiographic evidence, irrespective of clinical symptoms. The presence or absence of pain was not used as a defining criterion, and no stratification was performed between symptomatic and asymptomatic lesions. Similarly, this study did not distinguish between acute and chronic forms of apical periodontitis, as the design was not intended to capture temporal or clinical progression but rather susceptibility to radiographically defined disease.

Control individuals were defined as participants without radiographic evidence of periapical pathology in the examined teeth at the time of the evaluation. Teeth in the control group exhibited normal periapical bone architecture and absence of periapical radiolucency. Although the absence of a lifetime AP history could not be confirmed, the controls were classified as disease-free based on cross-sectional clinical and radiographic assessments.

### 2.5. Obtaining Genomic DNA

The study included 194 participants, comprising 60 cases clinically and radiographically diagnosed with AP and 134 controls with healthy periapical tissues and no history of endodontic pathology. AP was diagnosed based on clinical parameters (pain, tenderness, swelling, or sinus tract) and radiographic evidence of periapical radiolucency. Peripheral blood samples (5 mL) were collected by venipuncture in EDTA-containing tubes (Becton Dickinson, Franklin Lakes, NJ, USA). Genomic DNA was extracted using a silica-based spin column method (QIAamp DNA Mini Kit; Qiagen, Hilden, Germany) following the manufacturer’s instructions. DNA concentration and purity were assessed using spectrophotometry with a NanoDrop™ 2000 device (Thermo Fisher Scientific, Waltham, MA, USA), operated with NanoDrop™ 2000 software (v1.6).

### 2.6. Genotypic Analysis

Genotyping was performed using real-time polymerase chain reaction (qPCR) with TaqMan^®^ SNP Genotyping Assays (Applied Biosystems, Thermo Fisher Scientific, Foster City, CA, USA) for three variants: *MMP1* rs1799750 (−1607 1G/2G), *IL10* rs1800872 (−592 C>A), and *IL17A* rs7747909. Amplification and allelic discrimination were performed using a Rotor-Gene Q real-time PCR system (Qiagen, Hilden, Germany). The context sequences for each assay [VIC/FAM] are provided in Table 1; in these sequences, the first allele indicated within brackets corresponds to the VIC-labeled probe and the second to the FAM-labeled probe, according to the standard Thermo Fisher convention.

Each 10 µL reaction mixture contained 5.0 µL of TaqMan™ Genotyping Master Mix (Thermo Fisher Scientific, Waltham, MA, USA), 0.5 µL of the corresponding 20× SNP Genotyping Assay, 2.0 µL of genomic DNA (10–20 ng), and 2.5 µL of nuclease-free water (Thermo Fisher Scientific, Waltham, MA, USA). The thermal cycling conditions consisted of an initial denaturation step at 95 °C for 10 min, followed by 40 cycles of denaturation at 95 °C for 15 s and annealing/extension at 60 °C for 60 s. Allelic discrimination was performed using endpoint fluorescence cluster analysis with Rotor-Gene Q software (v2.3.5, Qiagen, Hilden, Germany).

### 2.7. Selection of Functional Polymorphisms

Three single-nucleotide polymorphisms (SNPs) from three genes involved in immune and inflammatory pathways were selected based on previous evidence of functional relevance and their reported associations with inflammatory regulation and host responses (Table 2). The selected markers included *MMP1* rs1799750 (−1607 1G/2G insertion/deletion), *IL10* rs1800872 (−592 C>A)**,** and *IL17A* rs7747909. These variants were selected because they modulate key mechanisms related to cytokine production, signal transduction, and transcriptional regulation of pro- and anti-inflammatory genes.

### 2.8. Replication and Functional Validation

Replication in an independent cohort and functional assays were not performed in this study. The investigation was conducted within the scope of a single-center dataset and was limited by the availability of biological material and ethical approval, which did not permit additional genotyping or laboratory testing. Therefore, this study is positioned as exploratory, and the identified associations should not be interpreted as definitive evidence of biological effects or clinical applicability. Future studies will require independent replication, ancestry-adjusted analyses, and functional assays (e.g., gene expression, cytokine quantification, and promoter characterization) to substantiate the preliminary findings reported in this study.

### 2.9. Statistical Analysis

All statistical analyses were conducted to evaluate the association between genetic variants and susceptibility to apical periodontitis (AP). Demographic and behavioral variables, including age, sex, oral hygiene practices, smoking status, and socioeconomic indicators, were not included as covariates in the genetic association models because these data were not systematically or consistently available for all participants in the original study protocol. Consequently, their inclusion in multivariate analyses would have introduced bias due to missing or heterogeneous data. Therefore, the analyses were restricted to unadjusted single-locus models, consistent with the exploratory nature of this candidate-gene study.

Data quality control procedures included verification of genotype call rates, assessment of Hardy–Weinberg equilibrium (HWE) in the control group, and comparison of minor allele frequencies (MAF) with reference data from the 1000 Genomes Project Admixed American (AMR) panel, including Colombians from Medellín (CLM) as a representative subpopulation. Genotype and allele frequencies were calculated by direct counting methods. HWE was evaluated using the chi-square (χ^2^) test with one degree of freedom (df). Descriptive statistics are presented as mean ± standard deviation (SD) or absolute and relative frequencies (%), as appropriate.

Genetic associations between single-nucleotide polymorphisms (SNPs) and AP were evaluated using a dominant inheritance model. This model was selected a priori based on biological plausibility, observed allele frequency distributions, and the need to ensure model stability, given the modest sample size. Recessive and additive genetic models were not evaluated because the number of minor allele homozygotes was insufficient to generate reliable estimates without increasing the risk of model instability or spurious associations. Effect sizes were expressed as odds ratios (ORs) with 95% confidence intervals (CIs). To account for multiple testing, *p*-values were adjusted using the Benjamini–Hochberg false discovery rate (FDR) procedure, with an adjusted *p*-value of <0.05 considered statistically significant. Odds ratios (ORs) and 95% confidence intervals (CIs) were estimated using unadjusted logistic regression models, according to the following equation:

$$\log((P(\mathrm{AP}))/(1-P(\mathrm{AP})))=\beta_{0}+\beta_{1}\times \mathrm{Genotype}$$

where AP denotes apical periodontitis status (coded as 1 for cases and 0 for controls), P(AP) represents the probability of apical periodontitis, $\beta_{0}$ is the intercept of the model, $\beta_{1}$ is the regression coefficient corresponding to the genotype effect, and Genotype is coded as 1 for carriers of at least one copy of the minor allele and 0 for homozygotes of the major allele.

No additional covariates were included in the model because demographic and behavioral variables were not consistently available for all participants.

Given the exploratory nature of this candidate gene-study, a post hoc power analysis was performed to estimate the ability of the study to detect genetic associations under the dominant inheritance model. Power calculations were based on standard assumptions for case–control genetic association studies, incorporating the observed sample size, minor allele frequencies, and a two-sided significance threshold of α = 0.05. Under these conditions, the study had adequate statistical power (>80%) to detect moderate-to-large effect sizes (odds ratio ≥ 2.5–3.0), whereas smaller genetic effects would not be reliably detected. Accordingly, the absence of an association for some variants should not be interpreted as evidence of no biological effect, but rather as a limitation of the statistical power inherent to the sample size. This analysis supports the interpretation of the present findings as exploratory and hypothesis-generating.

All analyses were conducted using R software (v4.3.1; R Foundation for Statistical Computing, Vienna, Austria) and SNPStats (https://www.snpstats.net/ accessed on 15 November 2025). Statistical significance was set at *p* < 0.05, unless otherwise specified.

## 3. Results

### 3.1. Study Population

A total of 194 participants were included, comprising 60 cases with AP and 134 controls without radiographic evidence of AP at the time of examination. Demographic variables such as age, sex, smoking status, oral hygiene habits, and socioeconomic factors were not consistently available and, therefore, were not included in the analysis.

Participants were genotyped for three single-nucleotide polymorphisms (SNPs) in immune-related genes: *MMP1* (rs1799750), *IL10* (rs1800872), and *IL17A* (rs7747909). The study population consisted of clinically confirmed cases of AP and controls without radiographic evidence of disease, with a mean age of 34.9 ± 12.6 years and a predominance of females (62%). Genotyping quality assessment demonstrated high technical performance, with no missing genotype calls across loci and an overall call rate of 100. Following a detailed evaluation of allelic discrimination plots and allele frequency plausibility, *MMP1* rs1799750, *IL10* rs1800872, and *IL17A* rs7747909 fulfilled all quality criteria and were retained for downstream association analyses.

**Figure 1:**
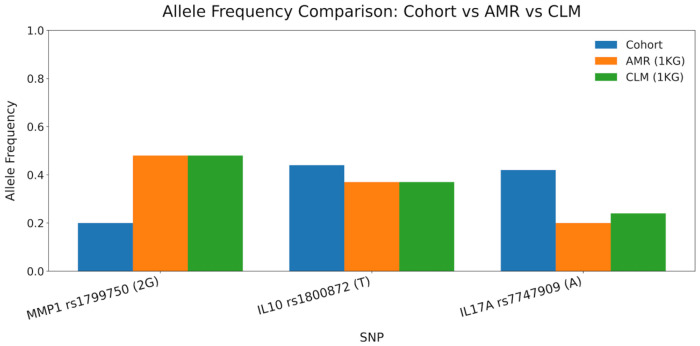
Allele frequency comparison between the cohort and 1000 Genomes AMR/CLM populations. Bar plot displaying allele frequencies for *MMP1* rs1799750 (2G), *IL10* rs1800872 (T), and *IL17A* rs7747909 (A). Values from the study cohort are shown along with reference frequencies from the Admixed American (AMR) and Colombian (CLM) populations of the 1000 Genomes Project. This comparison contextualizes the plausibility of allele distribution following genotyping QC.

The allele frequencies observed in the cohort for *MMP1* rs1799750, *IL10* rs1800872, and *IL17A* rs7747909 were compared with the reference values from the AMR and CLM populations of the 1000 Genomes Project. As shown in Figure 1, the frequencies of all three loci fell within the biologically plausible ranges for admixed Latin American populations, supporting the validity of the retained SNPs after QC. Allele frequencies in the overall cohort do not directly reflect the magnitude of the odds ratios, which depend on the *relative* distribution between cases and controls; therefore, AF differences by phenotype (not overall AF) account for the stronger association observed for *IL17A* rs7747909.

### 3.2. Genotyping and Hardy–Weinberg Equilibrium

All loci were genotyped using TaqMan^®^ allelic discrimination assays (Applied Biosystems, Foster City, CA, USA) on a Rotor-Gene Q Real-Time PCR System according to the manufacturer’s protocols. Genotyping success was 100% across all samples, with no missing data or ambiguous results. The Hardy–Weinberg equilibrium (HWE) was assessed in the control group for each variant using the χ^2^ test with one degree of freedom, and all loci conformed to the HWE expectations (*p* > 0.05). The observed minor allele frequencies (MAF) were consistent with those reported for the Admixed American (AMR) population from the 1000 Genomes Project, including Colombians from Medellín (CLM), confirming the representativeness of the study cohort within the regional genetic background.

### 3.3. Association Analysis of Genetic Polymorphisms

The genotype and allele frequencies of the three SNPs analyzed are summarized in Table 3. All loci were successfully genotyped with a 100% call rate, and the genotype distributions in the control group conformed to the HWE (*p* > 0.05). Only the inheritance models showing the most robust and statistically significant associations after FDR correction were retained for presentation.

Genotype and allele distribution analyses revealed strong and consistent associations between immune-related polymorphisms and AP susceptibility. All loci were successfully genotyped (100% call rate), and the genotype frequencies in the control group were in HWE (*p* > 0.05). Significant differences in genotype frequencies between cases and controls were identified for variants in *MMP1*, *IL10*, and *IL17A,* which remained statistically significant after Benjamini–Hochberg false discovery rate (FDR) correction.

Under the dominant genetic model, all three validated polymorphisms were significantly associated with AP risk. For *MMP1* rs1799750, carriers of at least one 2G allele demonstrated higher odds of disease relative to individuals with the 1G/1G genotype (OR = 3.47, 95% CI 1.40–8.58; *p* < 0.01; FDR-adjusted *p* = 0.013). Similarly, for *IL10* rs1800872, the presence of the T allele (CT + TT) was associated with increased susceptibility compared with the CC genotype (OR = 3.00, 95% CI 1.52–5.91; *p* < 0.001; FDR-adjusted *p* = 0.007). The strongest association was observed for *IL17A* rs7747909, where individuals carrying at least one A allele exhibited markedly greater odds of AP compared with GG homozygotes (OR = 8.95, 95% CI 3.61–22.15; *p* < 0.0001; FDR-adjusted *p* < 0.001). Collectively, these findings indicate that variations in inflammatory and matrix-remodeling pathways may influence susceptibility to disease, although these results remain exploratory, given the methodological constraints of the study.

## 4. Discussion

This study evaluated the association between three immune-related polymorphisms—*IL10* rs1800872, *MMP1* rs1799750, and *IL17A* rs7747909—and susceptibility to AP in a Colombian cohort, following rigorous quality control of genotyping. Although these variants represent biologically plausible candidates, the interpretation of these findings must be situated within the broader context of contemporary host genetic research on oral inflammatory diseases.

Over the last decade, large-scale genome-wide association studies (GWASs) have substantially reshaped our understanding of the genetic susceptibility to oral inflammation. Rather than identifying single variants with large effects, GWASs have consistently demonstrated a highly polygenic architecture, in which multiple loci exert modest effects and interact with environmental and behavioral exposures [10,11,12,13].

Genes involved in innate immune responses and extracellular matrix remodeling, such as *SIGLEC5*, *GLT6D1*, *DEFA1A3*, *VDR*, and the *IL1* cytokine cluster, have been repeatedly implicated in multi-cohort analyses, underscoring the central role of immune modulation in periodontal and periapical pathology [14]. In this context, the present findings support the concept of AP as a complex immunoinflammatory condition influenced by multiple genetic and non-genetic factors, rather than single variant effects.

The association observed for the *MMP1* promoter polymorphism rs1799750 (−1607 1G/2G) under a dominant model (OR = 3.47, 95% CI = 1.40–8.58; FDR = 0.013) is consistent with previous evidence linking this variant to increased transcriptional activity and enhanced collagenase-1 expression [25,26]. Elevated *MMP1* expression has been reported in periapical lesions and chronic periodontitis, and similar associations have been described in other inflammatory and degenerative conditions, including rheumatoid arthritis and osteoarthritis, where excessive matrix degradation contributes to tissue destruction [27,28]. Within the context of AP, this finding supports the role of extracellular matrix remodeling in lesion persistence and bone resorption.

Similarly, the *IL10* promoter variant rs1800872 (−592 C>T) was associated with an increased risk of AP (OR = 3.00, 95% CI = 1.52–5.91; FDR = 0.007). This variant has been linked to reduced promoter activity and lower IL-10 production through altered transcription factor binding [29,30]. Reduced *IL10*–mediated regulation may favor prolonged inflammatory responses, as suggested by studies on periodontitis and periapical lesions that reported associations between low-producer *IL10* genotypes and increased tissue breakdown [31]. These observations align with the role of *IL10* as a key regulator of inflammatory resolution in the oral tissues.

The strongest association in the present study was observed for *IL17A* rs7747909 (OR = 8.95, 95% CI = 3.61–22.15; FDR < 0.001). This intronic variant may influence *IL17A* regulation through post-transcriptional mechanisms or enhancer activity. Previous studies have shown that variants within the *IL17A* locus are associated with elevated cytokine levels and increased severity of chronic periodontitis and autoimmune diseases [32,33]. Consistently, elevated *IL17A* expression has been detected in periapical granulomas and cysts, promoting neutrophil recruitment and osteoclast-mediated bone resorption [34]. Although the magnitude of the observed effect should be interpreted cautiously, this association reinforces the relevance of Th17-related inflammatory pathways in AP susceptibility.

Despite these biologically plausible associations, several methodological considerations limit the causal inference. The present study did not include functional assays, such as gene expression analysis, cytokine profiling, or protein-level measurements, and therefore, cannot directly link the identified variants to altered biological activity in periapical tissues. The proposed mechanisms were inferred from the literature rather than experimentally demonstrated in this cohort, and the statistical associations do not imply causality.

From a broader perspective, the immunogenetic architecture of apical periodontitis is likely shaped not by isolated genetic variants but by the interactions among multiple genes involved in inflammatory signaling and tissue remodeling. In this context, epistatic interactions between *MMP1*, *IL10*, and *IL17A* are biologically plausible, as excessive extracellular matrix degradation, impaired anti-inflammatory regulation, and enhanced Th17-driven responses may act synergistically to promote chronic periapical inflammation. Although the present study was not powered to formally evaluate gene–gene interactions, this integrative framework underscores the need for future investigations using larger cohorts and systems-level approaches to disentangle epistatic effects on AP susceptibility.

Population ancestry is an important consideration. Latin American populations are characterized by complex three-way admixture involving Indigenous, European, and African ancestries, with marked regional heterogeneity [35,36]. Although the allele frequencies observed in this cohort were compatible with reference AMR populations, the absence of ancestry-informative markers or principal component adjustment precluded formal control of population stratification. Consequently, some of the observed associations may partially reflect the underlying population structure rather than disease-specific genetic effects.

Furthermore, evidence from meta-analyses indicates that associations identified in early candidate-gene studies frequently fail to replicate in larger, well-controlled cohorts due to limited sample sizes, population stratification, publication bias, and instability of effect size estimates [37]. These limitations are particularly relevant for immunogenetic traits influenced by multiple loci or environmental exposures. In line with this evidence, the present findings should be regarded as exploratory and hypothesis-generating, rather than confirmatory.

Finally, environmental and behavioral factors, including smoking, oral hygiene practices, dysbiosis, and socioeconomic conditions, are well-established modulators of the risk of oral inflammatory disease [38,39]. As these variables were not systematically collected, they could not be incorporated into the analytical models, further reinforcing the exploratory nature of these associations.

In summary, this study provides preliminary evidence suggesting that genetic variations in *IL10*, *IL17A*, and *MMP1* may contribute to susceptibility to AP in the Colombian population. However, these findings must be interpreted cautiously in light of the candidate-gene design, modest sample size, lack of ancestry adjustment, and absence of replication or functional validation. Future studies employing genome-wide approaches, polygenic analyses, ancestry-informed modeling, and mechanistic investigations are necessary to clarify the contribution of host genetics to apical periodontitis.

### Limitations and Future Directions

This study had several limitations that should be considered when interpreting the findings. First, the candidate gene design and modest sample size limit the statistical robustness of the analyses and increase the uncertainty of effect size estimates. The modest sample size limits the statistical power, particularly for detecting small genetic effects, and may contribute to unstable effect size estimates. Therefore, the associations reported herein should be interpreted as exploratory and hypothesis-generating rather than confirmatory.

Second, demographic, behavioral, and environmental variables, including age, sex, oral hygiene practices, smoking status, and socioeconomic factors, were not systematically or consistently available for all participants; therefore, they could not be incorporated into the adjusted genetic models. These variables are known to influence susceptibility to oral inflammatory diseases and may interact with the host genetic factors. Therefore, the reported associations should be interpreted as unadjusted and exploratory.

Third, AP was analyzed as a binary phenotype without stratification according to symptomatic status. Although symptomatic and asymptomatic forms of apical periodontitis may differ in terms of inflammatory burden, immune activation, and lesion dynamics, the available dataset did not provide sufficient statistical power to evaluate symptom-specific genetic associations. Consequently, it is possible that distinct genetic determinants contribute to disease susceptibility, lesion persistence, and symptom expression.

Additionally, controls were defined cross-sectionally and lacked information on lifetime periapical history, which may have introduced nondifferential misclassification and biased effect estimates toward the null hypothesis. No independent replication cohort was available, and no functional assays, such as gene expression analysis, cytokine quantification, promoter activity assessment, or protein-level studies, were performed. Therefore, the associations identified in this study cannot establish a biological causality. In addition, genetic associations were evaluated exclusively under a dominant inheritance model; alternative genetic models could not be robustly assessed because of limited genotype counts.

An additional important limitation of the present study is the absence of microbiological characterization. AP is fundamentally a host–microbiota disease in which the composition, functional potential, and ecological organization of the root canal and periapical microbiome play critical roles in disease initiation, persistence, and treatment response [40]. In this context, host genetic susceptibility is likely to modulate immune responses to specific microbial communities, rather than acting independently. Because microbial sampling and sequencing were not included in the original study design, it was not possible to evaluate host–microbiome interactions or determine whether the observed genetic associations were contingent on specific microbial profiles. Recent evidence emphasizes that AP is associated with distinct microbial signatures in primary and post-treatment lesions, as well as functional shifts in microbial communities that influence the inflammatory burden and tissue destruction. Consequently, the absence of microbiome data limits the biological resolution of the present findings and precludes an integrative interpretation at the host–microbial interface.

Future studies should adopt integrative, multi-layered approaches combining host genetic data with high-resolution microbial profiling, such as 16S rRNA gene sequencing, shotgun metagenomics, or metatranscriptomics, to elucidate how host immunogenetic variation shapes microbial community structure and function in AP. Such approaches, as recently proposed in conceptual and systematic frameworks for AP research, are essential to move beyond single-layer association studies toward a systems-level understanding of disease pathogenesis.

Taken together, these limitations indicate that the present findings should be considered strictly exploratory and hypothesis-generating. Future studies incorporating larger ancestry-characterized cohorts, comprehensive phenotypic and environmental data, longitudinal clinical characterization, and functional validation are required to clarify the immunogenetic mechanisms underlying susceptibility to AP.

## Figures and Tables

**Table 1:** TaqMan^®^ genotyping assays and context sequences for immune-related SNPs were analyzed.

Gene	rsID	Assay ID TaqMan^®^	Context Sequence [VIC/FAM] (5′ → 3′)
*IL17A*	rs7747909	C__29315993_10	AATTAAAGCTTCAGAGGTAACACTT [A/G]GCCAAGATATGAGATCTGAATTACC
*IL10*	rs1800872 (−592 C>A)	C___1747363_10	CTTTCCAGAGACTGGCTTCCTACAG [T/G]ACAGGCGGGGTCACAGGATGTGTTC
*MMP1*	rs1799750 (−1607 1G/2G)	C__34384693_10	TGGATTGATTTGAGATAAGTCATAT [C/−]CTTTCTAATTATTTAACTACAATTT

**Table 2:** Molecular characteristics and predicted functional effects of immune-related SNPs analyzed in this study.

Gene	SNP	Name/Functional Location	Alleles (Ref/Alt)	Base Changes (Context Sequence 5′ → 3′)	Functional Effect/Consequence	Region	MAF (1000G AMR)
*MMP1*	rs1799750	−1607 1G/2G (promoter Ins/Del)	−/G	TGGATTGATTTGAGATAAGTCATAT [C/−]CTTTCTAATTATTTAACTACAATTT	Creates additional binding site; increases *MMP1* transcription	Promoter	2G = 0.32
*IL10*	rs1800872	−592 C>A (promoter variant)	C/A	CTTTCCAGAGACTGGCTTCCTACAG [T/G]ACAGGCGGGGTCACAGGATGTGTTC	Modulates *IL10* promoter activity; impacts anti-inflammatory cytokine expression	Promoter	A = 0.21
*IL17A*	rs7747909	Intronic variant	A/G	AATTAAAGCTTCAGAGGTAACACTT [A/G]GCCAAGATATGAGATCTGAATTACC	Potential regulatory variant influencing *IL17A* splicing or expression	Intron 1	G = 0.29

MAF: Minor allele frequency (AMR population, 1000 Genomes Project).

**Table 3:** Association between candidate gene polymorphisms and susceptibility to apical periodontitis under a dominant genetic model.

Gene	SNP (rsID)	Genetic Model	Genotype Comparison	OR (95% CI)	Raw *p*-Value	FDR-Adjusted *p*-Value
*MMP1*	rs1799750	Dominant	1G/2G + 2G/2G vs. 1G/1G	3.47 (1.40–8.58)	0.007	0.013
*IL10*	rs1800872	Dominant	CT + TT vs. CC	3.00 (1.52–5.91)	0.003	0.007
*IL17A*	rs7747909	Dominant	AG + GG vs. AA	8.95 (3.61–22.15)	<0.001	<0.001

Odds ratios (ORs) and 95% confidence intervals (CIs) were estimated using unadjusted logistic regression models under a dominant inheritance model, in which carriers of at least one copy of the minor allele were compared with homozygous carriers of the major allele. No adjustment for demographic or behavioral covariates was performed because these variables were not systematically available for all the participants. Raw *p*-values were adjusted for multiple tests using the Benjamini–Hochberg false discovery rate (FDR) procedure.

## Data Availability

The data presented in this study are contained within this article. Further inquiries can be directed to the corresponding author.

## Abbreviations

AP	Apical periodontitis
AMR	Admixed American
CI	Confidence interval
CLM	Colombians from Medellín
FDR	False discovery rate
GWAS	Genome-wide association study
HWE	Hardy–Weinberg equilibrium
IL	Interleukin
MAF	Minor allele frequency
MMP	Matrix metalloproteinase
OR	Odds ratio
PCA	Principal component analysis
qPCR	Quantitative polymerase chain reaction
SNP	Single-nucleotide polymorphism
